# Identification of immune landscape signatures associated with clinical and prognostic features of hepatocellular carcinoma

**DOI:** 10.18632/aging.103977

**Published:** 2020-10-13

**Authors:** Hongmei Yan, Yuchuan Chen, Kai Wang, Lu Yu, Xixin Huang, Qianyu Li, Yuwen Xie, Jiayu Lin, Yueyun He, Xinyu Yi, Yanzhi Wang, Longhua Chen, Yi Ding, Yiyi Li

**Affiliations:** 1 Department of Radiation Oncology, Nanfang Hospital, Southern Medical University, Guangzhou 510515, China; 2 Division of Hepatobiliopancreatic Surgery, Department of General Surgery, Nanfang Hospital, Southern Medical University, Guangzhou 510515, China; 3 The First School of Clinical Medicine, Southern Medical University, Guangzhou 510515, China; 4 Medical Imaging Specialty, the First School of Clinical Medicine, Southern Medical University, Guangzhou 510515, China; 5 Department of Pathology, Nanfang Hospital, Southern Medical University, Guangzhou 510515, China; 6 Clinical Medicine Specialty, the First School of Clinical Medicine, Southern Medical University, Guangzhou, 510515, China

**Keywords:** hepatocellular carcinoma, heterogeneity, immunity, tumor microenvironment, immunotherapy

## Abstract

While cancer immunotherapy has been remarkably successful in some malignancies, some cancers derive limited benefit from current immunotherapies. Here, we combined immune landscape signatures with hepatocellular carcinoma clinical and prognostic features to classify them into distinct subtypes. The immunogenomic profiles, stromal cell features and immune cell composition of the subtypes were then systematically analyzed. Two independent prognostic indexes were established based on 6 immune-related genes and 17 differentially expressed genes associated with stromal cell content. These indexes were significantly correlated with tumor mutation burden, deficient DNA mismatch repair and microsatellite instability. In addition, tumor-infiltrating lymphocytes, including activated NK cells, resting memory CD4 T-cells, eosinophils, and activated mast cells were significantly correlated with hepatocellular carcinoma survival. In conclusion, we have comprehensively described the immune landscape signatures and identified prognostic immune-associated biomarkers of hepatocellular carcinoma. Our findings highlight potential novel avenues for improving responses to immunotherapy.

## INTRODUCTION

Hepatocellular carcinoma (HCC) is ranked as the 3^rd^ leading cause of cancer deaths globally [1]. Most HCCs are associated with liver cirrhosis arising from chronic infection of hepatitis B and C viruses. Liver cirrhosis is also caused by alcohol consumption and fatty liver disease [2]. Treatment strategies for HCC are dependent on disease stage. Surgery is the standard treatment for early stage HCC and has a 70% 5-year survival rate. Where surgery or liver transplantation is unfeasible, loco-regional therapies, including radiotherapy, radiofrequency, thermal and non-thermal ablation, and trans-arterial chemoembolization may be recommended as second line therapies. However, the 3-5-year survival rates of such approaches are highly variable [3]. For advanced unresectable HCC, the recommended treatments include regorafenib and sorafenib (tyrosine kinase inhibitors (TKIs)) and the vascular endothelial growth factor receptors 1 through 3 (VEGFR1-3) inhibitor, lenvatinib. However, these therapies offer very limited survival benefit [4].

Cancer immunotherapy has emerged as one of the most promising cancer treatments for various types of advanced solid tumors, including HCC [5]. Several immune-based therapies such as peptide vaccines against HCC antigens, natural killer cell therapies, and chimeric antigen receptor-engineered T cell therapies are under investigation in phase I/II trials [5]. Among them, immune checkpoint inhibitors have shown good prospects in the treatment of advanced HCC as evidenced by durable objective response rates (ORRs) and acceptable safety profiles in phase I/II trials [6].

While immunotherapy is remarkably successful in many HCC cases, it remains unclear why a subset of HCC patients fails to respond to it. Intratumoral genetic heterogeneity is a well-known feature of cancer [7]. Tumor mutation burden (TMB), neoantigen load, programmed cell death ligand 1 (PD-L1) levels, impaired DNA mismatch repair (MMR), and microsatellite instability (MSI) have been found to affect response of cancer cells to immunotherapy [8, 9]. Additionally, the highly heterogeneous immune microenvironment has been associated with various aspects of cancer [10]. For instance, non-tumor cell types within or around tumors, including stromal and immune cells, also influence cancer progression [11]. Thus, development of potent immunotherapy requires a comprehensive understanding of the heterogeneity of tumor and tumor immune microenvironment (TIME).

In the present study, HCC tissues were subclassified on the basis of immunogenomic profiles, stromal cell features, and immune cell composition. We systematically analyzed the molecular features of each sub-class, such as gene ontology, genes, chemotactic factors, regulatory pathways and networks, and integrated them with various clinicopathological features and patient clinical outcomes. Our findings highlight the potential clinical utility of individualized immune signatures in prognostic stratification and personalized immunotherapy for HCC patients.

## RESULTS

### Immunogenomic profiling identifies 2 HCC subtypes

**Figure 1:**
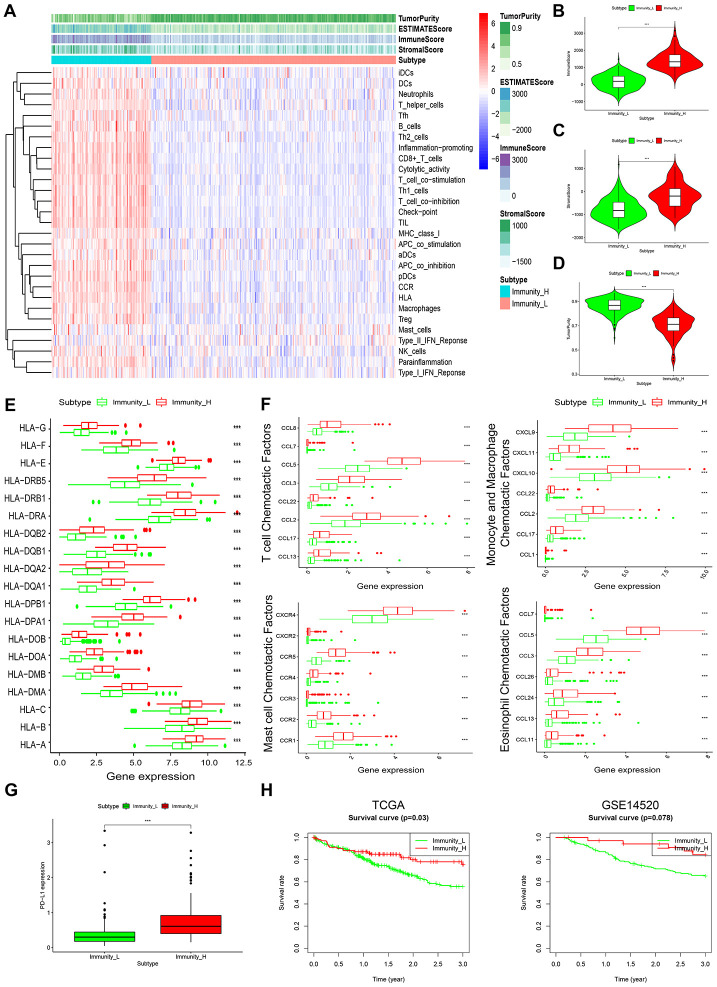
**Characterization of 2 HCC subtypes based on immunogenomic profiling.** (**A**) Heatmap of normalized ssGSEA scores for tumor purity, ESTIMATE score, Immune Score, and Stromal Score. Tumor samples were grouped into 2 immune classes, Immunity_H and Immunity_L, based on 29 immune-associated gene sets. (**B**–**D**) Violin plot analysis comparing the Immune Score (**B**), Stromal Score (**C**) and tumor purity (**D**) between Immunity_H and Immunity_L (Mann-Whitney U test). (**E**) Box plot comparison of relative HLA gene expression between HCC subtypes (T-test). (**F**) Box plots comparing expression levels of various immunologic activity related chemotactic factors between HCC subtypes. (**G**) Comparison of PD-L1 expression in Immunity_H and Immunity_L (T-test). (**H**) Comparison of 3-year survival between HCC subtypes in TCGA and GSE14520 datasets (log-rank test).

Enrichment level and activity of several immune cells, pathways or functions in HCC were analyzed using single sample gene-set enrichment analysis (ssGSEA) score based on 29 immune-associated gene sets [12, 13]. According to the ssGSEA score of these gene sets, we hierarchically clustered 369 HCC samples from TCGA datasets. Consequently, 2 distinct clusters, termed Immunity_L (Immunity Low) and Immunity_H (Immunity High) were identified (Figure 1A). Lower immune scores were seen in Immunity_L than in Immunity_H (Figure 1B). Notably, analysis of tumor purity and stromal score revealed opposite results, with tumor purity being low in Immunity_H and high in Immunity_L, while stromal score was high in Immunity_H and low in Immunity_L (Figure 1C, 1D). This suggests that Immunity_L group harbors more tumor cells whereas Immunity_H harbors more stromal and immune cells.

It was seen that most HLA (human leukocyte antigen) genes were upregulated in Immunity_H than in Immunity_L (Figure 1E). Levels of various immunologic chemotactic factors were also markedly elevated in Immunity_H, including T-cell, monocyte, macrophage, mast cell and eosinophil chemotactic factors (Figure 1F) [14]. Evaluation of PD-L1 expression in both HCC subtypes showed that PD-L1 levels were markedly higher in Immunity_H than in Immunity_L (Figure 1G). This implies that the Immunity_H subtype may show a stronger response to anti-PD-L1 immunotherapy given that PD-L1 expression positively associates with immunotherapeutic responsiveness [15].

Survival analyses of 2 public datasets showed that the 2 HCC subtypes were associated with different clinical outcomes. The Immunity_H subtype corresponded with significantly better 3-year survival relative to Immunity_L (Figure 1H), which is consistent with previous findings in which HCC associated with higher immune activity exhibited better clinical outcomes [16]. These data highlight the value of subtyping as the basis of immunogenomic profiling.

### Changes in TIME and prognosis based on differential immune-related genes

### Identification of HCC subtype-specific gene expression profiles

**Figure 2:**
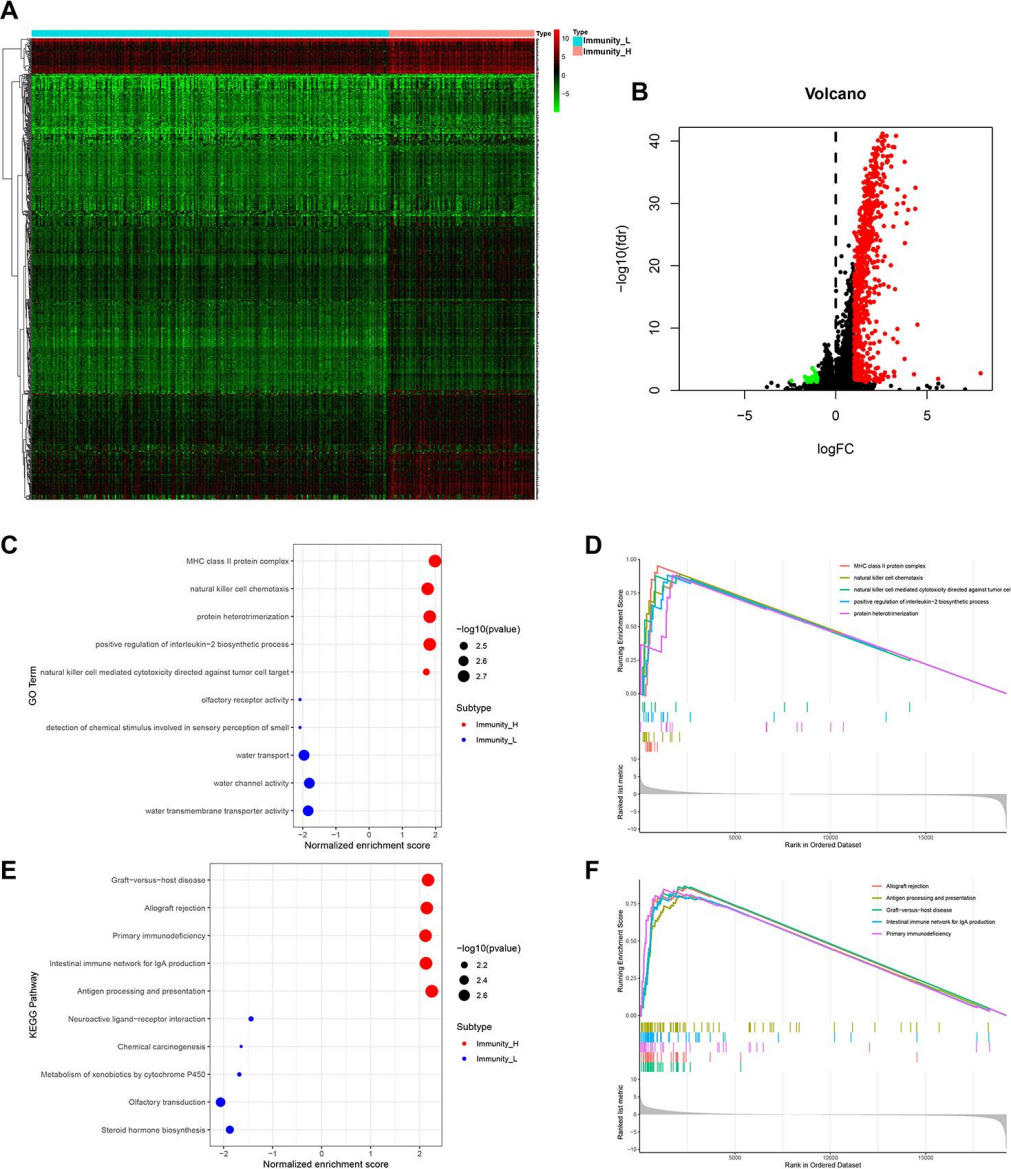
**HCC subtype-specific gene expression profiles.** (**A**, **B**) Heatmap and volcano plot of differentially expressed genes between Immunity_H and Immunity_L. Red, green and black dots indicate upregulated, downregulated and unchanged genes. It also applies to the following figures. (**C**–**F**) Significantly enriched GO terms (**C**, **D**) and KEGG pathways (**E**, **F**) of HCC subtypes.

We then used EdgeR to identify HCC subtype-specific gene expression profiles [17]. A total of 729 genes were differentially expressed between Immunity_H and Immunity_L subtypes. Among these, 706 were upregulated and 23 were downregulated (Figure 2A, 2B). GO term analysis revealed that MHC-class II protein complex, natural killer cell chemotaxis and positive regulation of interrleukin-2 biosynthetic process were the most significantly enriched cellular components and biological process in Immunity_H (Figure 2C, 2D). KEGG pathway analysis identified allograft rejection, antigen presentation and processing to be most highly enriched in Immunity_H (Figure 2E, F). These genetic alterations affect the immune system modulations [18–21].

### Identification of differentially expressed immune-related genes

**Figure 3:**
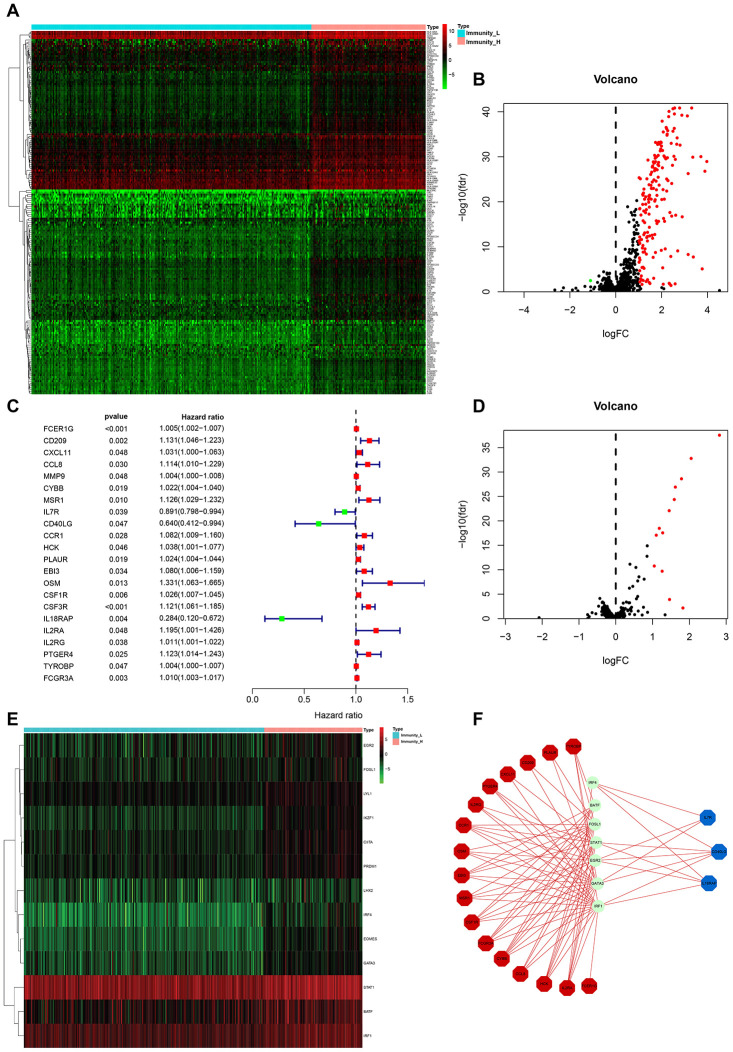
**HCC subtype-specific differentially expressed IRGs.** (**A**, **B**) Heatmap and volcano plot of the differentially expressed IRGs between Immunity_H and Immunity_L. (**C**) Forest plot of hazard ratios and corresponding 95% confidence intervals were estimated from univariate Cox’s regression analyses. Variables significantly associated with a good and poor OS are shown in green and red, respectively. (**D**, **E**) Volcano plot and heatmap of the differentially expressed TF genes between Immunity_H and Immunity_L. (**F**) Combinatorial TFs-IRGs regulation networks. Red, blue, and green, indicate high risk genes, low risk genes, and TF genes. It also applies to the following figures.

From the above set of genes, we identified 181 differentially expressed immune-related genes (IRGs) between Immunity_H and Immunity_L, of which 180 were upregulated and 1 was downregulated (Figure 3A, 3B). To identify the IRGs actively participating in HCC tumorigenesis and progression, we selected 22 differentially expressed IRGs that showed marked correlation with clinical outcomes. A forest plot of hazard ratios revealed 3 of these IRGs as protective factors, and the remaining 19 as predictors of poor prognosis (Figure 3C). To elucidate the molecular mechanisms underlying the clinical effects of the 22 IRGs, we evaluated the expression patterns of 318 transcription factors (TFs). Consequently, 13 TFs were found to be substantially higher in Immunity_H than in Immunity_L (Figure 3D, 3E). A regulatory network was established using the IRGs and TFs. A TF-based regulatory schematic was used to show the regulatory relationships between 7 of the 13 TFs and 20 of the 22 prognosis-associated IRGs (Figure 3F).

### Evaluation of clinical outcomes based on the prognostic IRG panel

**Figure 4:**
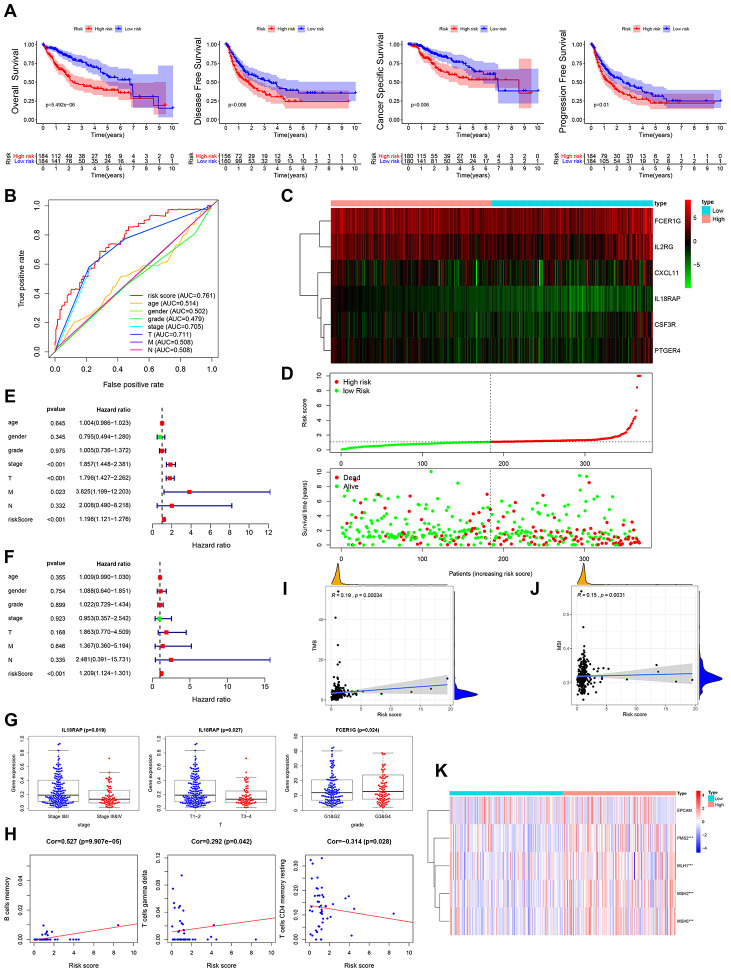
**Clinical utility of prognostic IRG panel and IRGs-based prognostic index.** (**A**) Overall survival, disease-free survival, cancer-specific survival, and progression-free survival of patients between high- and low-risk groups based on prognostic IRGs. (**B**) ROC curve analysis of risk score compared with clinicopathological features (age, gender, pathologic grade, clinical stage, tumor stage, lymph node metastasis and distant metastasis). (**C**, **D**) Differential risk scores, survival status and expression pattern of 6 IRGs in HCC patients. (**E**, **F**) Univariate and multivariate Cox regression analysis showing the independent prognostic value of this risk score. (**G**) Relationships between genes in the prognostic IRG panel and clinicopathological characteristics of HCC patients. (**H**) Pearson correlation analysis between risk score and infiltration abundances of immune cells. (**I**) Spearman correlation analysis between risk score and tumor mutation burden (TMB). (**J**) Spearman correlation analysis between risk score and microsatellite instability (MSI). (**K**) Heatmap visualization of the expression of 4 DNA mismatch repair (MMR) genes related to risk score. *** *p =* <0.001.

Multivariate Cox regression analysis was performed to assess the risk score for the prognosis-associated IRGs and constructed a prognostic signature for stratification of HCC patients into 2 groups based on the clinical outcomes. Disease-free survival, overall survival, cancer specific survival and progression-free survival were higher in the low risk group than in the high risk group (Figure 4A). The area under the curve (AUC) of the survival-dependent receiver operating characteristic (ROC) curve was 0.761, which was higher than that of clinicopathologic factors, indicating a high prognostic power for this IRG panel (Figure 4B). Heatmaps were established to visualize the expression profile of the 6 genes included in this panel for the low and high risk groups (Figure 4C). The risk score was calculated as follows: [Expression level of IL18RAP * (-2.5273)] + [Expression level of FCER1G * 0.0038] + [Expression level of CXCL11 * 0.0340] + [Expression level of CSF3R * 0.1133] + [Expression level of IL2RG * 0.0138] + [Expression level of PTGER4 * 0.1130]. This analysis showed that high risk scores corresponded with high fatality (Figure 4D). Univariate and multivariate Cox regression analyses revealed that the risk score could independently predict the prognosis of patients after adjusting for other parameters like distant metastasis, lymph node metastasis, tumor stage, clinical stage, pathologic grade, age, and gender (Figure 4E, 4F). Thus, the IRGs-based prognostic index may help stratify HCC patients based on expected clinical outcomes.

### Clinical utility of the prognostic IRG panel and IRGs-based prognostic index

In further analysis, we examined the relationship between genes in the prognostics panel and clinicopathological features of HCC patients. This analysis revealed a significant negative correlation between IL18RAP and distant metastasis, advanced T stage and advanced clinical stage (Figure 4G, Supplementary Figure 1A). FCER1G exhibited significant positive correlation with poor pathologic grade (Figure 4G). CXCL11 showed significant negative correlation with lymph node metastasis and distant metastasis (Supplementary Figure 1A). CSF3R exhibited a marked negative correlation with distant metastasis (Supplementary Figure 1A). IL2RG exhibited significant negative correlation with lymph node metastasis (Supplementary Figure 1A). To determine if the IRGs-based prognostic index accurately reflects tumor immune microenvironment status, we examined the association of risk scores with infiltration abundance of various types of immune cells. Significant positive correlation was observed between IRGs-based prognostic index and infiltration abundance of memory B-cells or gamma delta T-cells (r=0.527, *p=*<0.001; r=0.292, *p=*<0.05) (Figure 4H). Negative correlation was observed between the IRGs-based prognostic index and infiltration abundance of resting memory CD4 T-cells (r=-0.314, P<0.05) (Figure 4H).

Tumor mutation burden (TMB), impaired DNA mismatch repair (MMR), and microsatellite instability (MSI) have been found to affect response to cancer immunotherapy. Interestingly, we find that the prognostic IRG panel-based risk score correlates positively with TMB and MSI (Figure 4I, 4J). Relative to the low risk group, PMS2, MLH1, MSH2 and MSH6 (the MMR genes) were significantly elevated in the high-risk group (Figure 4K). These results strongly suggest that patients in high risk group may benefit from immunotherapy.

### Changes in TIME and prognosis based on differential stromal cell infiltration

### Re-subtyping HCC according to differential stromal components

**Figure 5:**
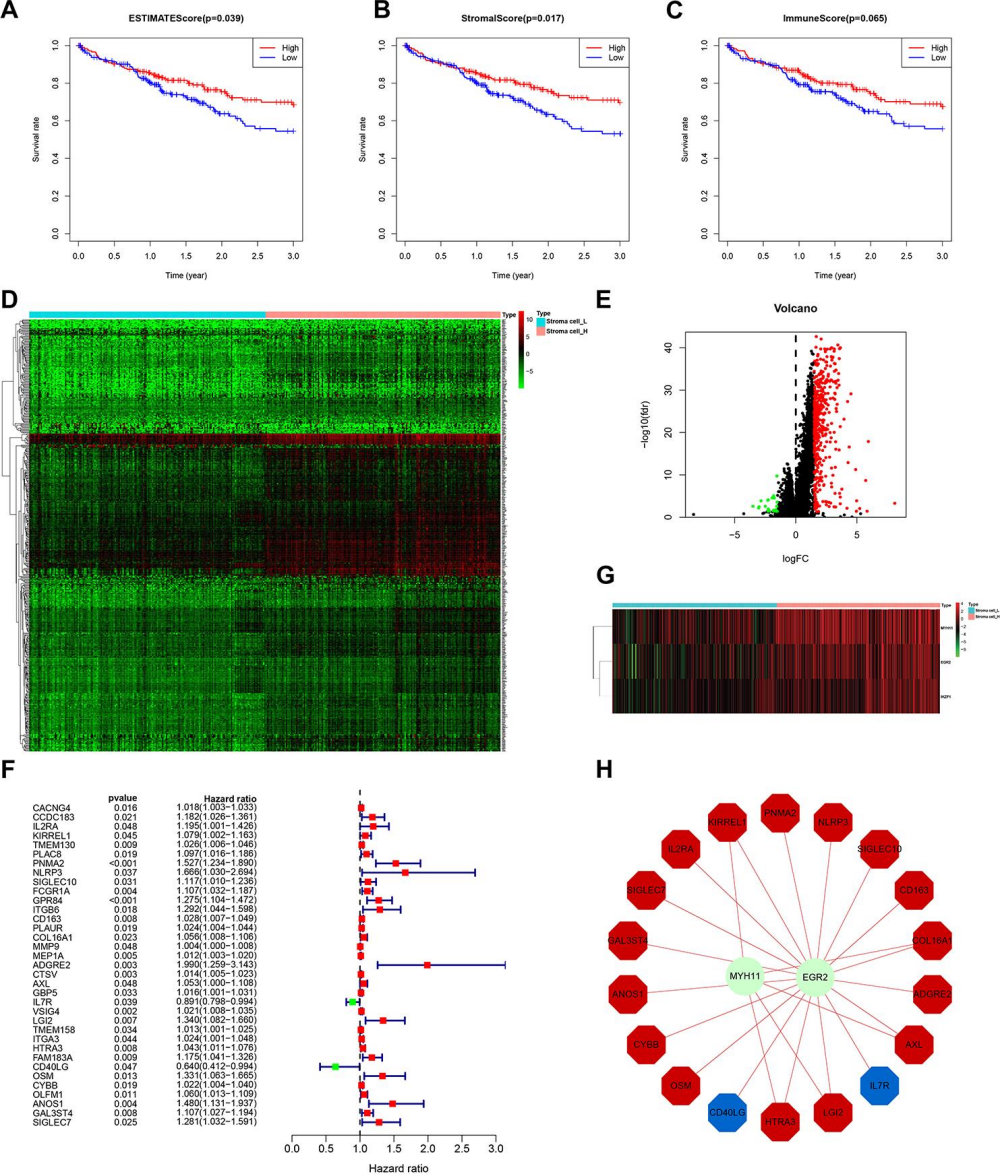
**Identification of stromal cell content-related prognostic genes.** (**A**–**C**) Comparison of 3-year survival between Stroma_H and Stroma_L, Stroma cell_H and Stroma cell_L, and Immune cell_H and Immune cell_L. (**D**, **E**) Heatmap and volcano plot of DEGs between Stroma cell_H and Stroma cell_L. (**F**) Forest plot of hazard ratios and corresponding 95% confidence intervals estimated from univariate Cox regression analyses. Variables significantly associated with good and poor OS are shown in green and red, respectively. (**G**) Heatmap of the differentially expressed TF genes between Stroma cell_H and Stroma cell_L. (**H**) Combinatorial TF regulatory networks.

Although ssGSEA-based HCC subtyping showed that Immunity_H contains more stromal and immune cells (Figure 1B, 1C), a more rigorous grouping method based on ESTIMATE scores (stromal score combined with immune score) is necessary to determine disease features related to stromal contents. Thus, we reclassified the 369 HCC samples into high stromal content group (Stroma_H) and low stromal content group (Stroma_L) based on median ESTIMATE score. Expectedly, survival analysis suggested that Stroma_H correlated with significantly better 3-year survival than Stroma_L (Figure 5A). To identify the stromal components responsible for distinct clinical outcomes, we further divided the samples into the high stromal cell content group (Stroma cell_H), low stromal cell content group (Stroma cell_L), high immune cell content group (Immune cell_H) and low immune cell content group (Immune cell_L), based on median value of stromal score and immune score, respectively. Survival analyses indicated that Stroma cell_H significantly correlated with better 3-year survival, while the effect of immune cell content on survival rate did not significantly differ between groups (Figure 5B, 5C).

### Identification of stromal cell content-related prognostic genes

To identify stromal cell content-related differentially expressed genes (DEGs), we compared Stroma cell_H gene expression profiles to those of Stroma cell_L and identified 480 significantly associated genes. Of these, 455 genes were elevated and 25 genes were suppressed in Stroma cell_H (Figure 5D, 5E). 35 prognostic DEGs significantly associated with favorable or unfavorable HCC survival outcomes were then identified from the above DEGs. A forest plot of hazard ratios showed that 2 of these DEGs were protective factors and 33 were significantly correlated with poor prognosis (Figure 5F). To explore the potential regulatory mechanisms of our prognostic DEGs, we evaluated the expression profiles of the 318 TFs and found 3 to be significantly elevated in Stroma cell_H relative to Stroma cell_L (Figure 5G). The regulatory network analysis showed relationships between 2 of the 3 TFs and 18 of the 35 prognostic DEGs (Figure 5H).

### Evaluation of clinical outcomes based on prognostic DEG panel

**Figure 6:**
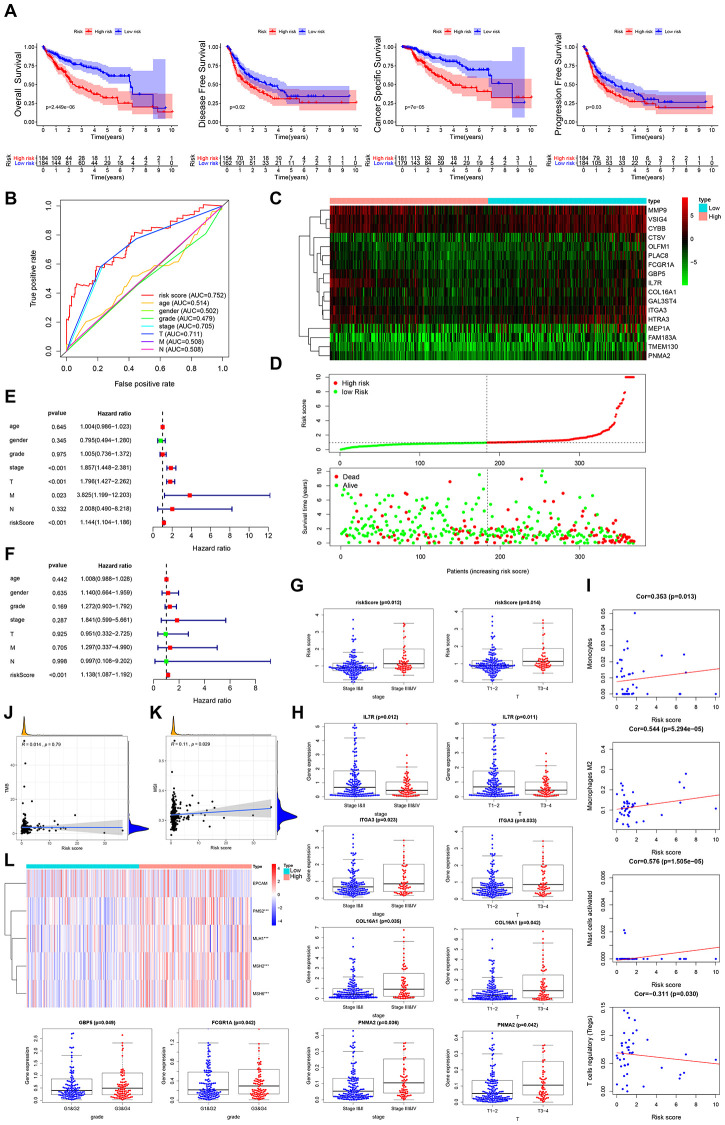
**Clinical utility of stromal cell content-related prognostic DEG panel and stromal score.** (**A**) Overall survival, disease-free survival, cancer-specific survival, and progression-free survival of patients between high- and low-risk groups based on stromal cell content-associated prognostic genes. (**B**) ROC curve analysis of risk score relative to clinicopathological features (age, gender, pathologic grade, clinical stage, tumor stage, lymph node metastasis and distant metastasis). (**C**, **D**) Differential risk scores, survival status and expression pattern of 17 IRGs in HCC patients. (**E**, **F**) Univariate and multivariate Cox regression analysis showing the independent prognostic value of this risk score. (**G**) Relationship between risk score and the clinical stage, as well as the T stage of HCC patients. (**H**) Relationships between genes in the prognostic DEG panel and the clinicopathological characteristics of HCC patients. (**I**) Pearson correlation analysis between the stromal score and infiltration abundances of immune cells. (**J**) Spearman correlation analysis between the risk score and TMB. (**K**) Spearman correlation analysis between the risk score and MSI. (**L**) Heatmap visualization of the expression of 4 DNA MMR genes related to the risk score. *** *p* = <0.001.

Next, we assessed the risk score for stromal cell content-related prognostic genes and designed a prognostic signature to subcategorize HCC patients into 2 groups based on prognosis. This analysis found that cancer specific survival, disease-free survival, overall survival, and progression-free survival were higher in the low risk group than in the high risk group (Figure 6A). The AUC of the survival-dependent ROC curve was 0.752, which is higher than the AUCs of clinicopathologic factors, suggesting high prognostic value for this DEG panel (Figure 6B). Heatmaps were used to visualize expression profiles of the 17 genes in the panel from low risk to high risk group (Figure 6C). Risk score was calculated as follows: [Expression level of IL7R * (-0.2979)] + [Expression level of FCGR1A * (-0.2721)] + [Expression level of GAL3ST4 * (-0.2270)] + [Expression level of COL16A1 * (-0.0941)] + [Expression level of ITGA3 * (-0.0522)] + [Expression level of MMP9 * (-0.0144) + [Expression level of CTSV * 0.0166]+[Expression level of MEP1A* 0.0177]+[Expression level of GBP5 * 0.0192]+[Expression level of TMEM130 * 0.0474]+[Expression level of VSIG4 * 0.0873]+[Expression level of CYBB * 0.0882]+[Expression level of HTRA3 * 0.1048]+[Expression level of PLAC8 * 0.1050]+[Expression level of OLFM1 * 0.1162]+[Expression level of FAM183A * 0.1604]+[Expression level of PNMA2 * 0.4091]. High risk scores correlated with higher contemporaneous deaths (Figure 6D). Furthermore, the risk score exhibited independent prognostic potential upon adjustment of other parameters, like distant metastasis, lymph node metastasis, tumor stage, clinical stage, pathologic grade, gender and age (Figure 6E, 6F). Stromal cell content-related, DEG-based prognostic index, exhibited significant positive correlation with advanced clinical stage and advanced T stage (Figure 6G).

### Clinical utility of the prognostic DEG panel and stromal score

Analysis of the relationships between the above genes in DEG panel and HCC clinicopathological features revealed that GBP5, IL7R, PNMA2, ITGA3, COL16A1, FCGR1A, MEP1A, CTSV, FAM183A and PLAC8 expression were strongly linked to distant metastasis, lymph node metastasis, T stage, clinical stage or pathologic grade (Figure 6H, Supplementary Figure 1B). Moreover, analysis of the relationships between stromal cell content-related DEGs-based prognostic index and infiltration abundance of various immune cell types revealed a significant positive correlation between stromal cell content-related, DEG-based prognostic index and infiltration by monocytes, M2 macrophages, or activated mast cells (r=0.353, *p=*<0.05; r=0.544, *p=*<0.001; r=0.576, *p=*<0.001) (Figure 6I). A negative correlation was observed between the stromal cell content-related, DEGs-based prognostic index, and infiltration abundances of regulatory T-cells (r=-0.314, *p=*<0.05) (Figure 6I). Furthermore, we found that risk score based on the stromal cell content-related prognostic genes positively correlated with MSI (Figure 6K), but did not significantly correlate with TMB (Figure 6J). Relative to the low risk group, expression of the MMR genes PMS2, MLH1, MSH2, and MSH6, was higher in the high risk group (Figure 6L), indicating that patients in the high-risk group derived much benefit from immunotherapy.

### Changes in TIME and prognosis based on differential immune cell composition

### Identification the heterogeneous of tumor-infiltrating immune cells

**Figure 7:**
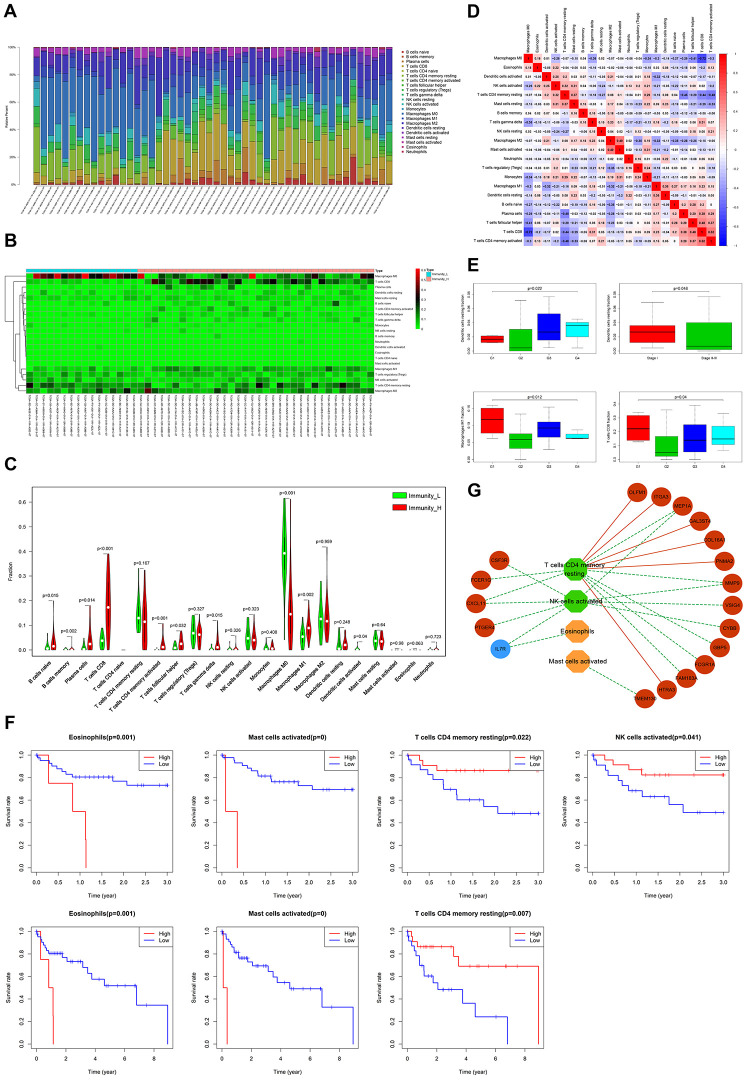
**Evaluation of clinical outcomes based on differential TIICs.** (**A**) Relative proportions of 22 immune cell subpopulations in HCC patients. (**B**) Heatmap visualization of differential immune cell proportions between Immunity_H and Immunity_L. (**C**) Violin plot analysis exhibiting distinct immune cells subpopulation between Immunity_H and Immunity_L. (**D**) Correlation matrix of all 22 immune cell proportions in HCC. (**E**) Box plot depicting relationships between TIICs and pathologic grade, as well as the clinical stage (Kruskal-Wallis tests). (**F**) Kaplan-Meier survival curves show the relationship between TIICs and survival. (**G**) The regulation networks among prognostic TIICs, IRG panel, and stromal cell content-related DEG panel. Green and blue represent protective factors. Red and orange represent the opposite.

Next, we used CIBERSORT [22], to investigate immune infiltration of 22 subpopulations of immune cells in the above HCC samples. Fifty cases with reliably predicted results were identified using a p-value cutoff of *≤*0.05. The proportions of immune cells in HCC vary significantly between samples (Figure 7A). Since we speculated that variation in the proportion of tumor-infiltrating immune cells (TIICs) may represent differences between subtypes, the samples of Immunity_H (34/50 cases) and Immunity_L (16/50cases), were separated into 2 discrete groups for further investigation (Figure 7B). Relative to Immunity_L, naive B-cells, memory B-cells, plasma cells, CD8 T-cells, activated CD4 memory T-cells, follicular helper T-cells, gamma delta T-cells, M0 macrophages, M1 macrophages and activated dendritic cells, presented significant abundance differences in Immunity_H (Figure 7C). The proportions of different TIIC subpopulations exhibited only weakly-moderately correlation with each other (Figure 7D). These results indicate the clinical significance of heterogeneous immune infiltration in HCC.

### Evaluation of clinical outcomes based on differential TIICs

The presence of tumor-infiltrating lymphocytes (TILs) in TIICs is associated with better responses to immunotherapy and improved clinical outcomes [23]. Expectedly, resting dendritic cells were strongly associated with higher pathologic grade and lower stage, whereas M1 macrophages and CD8 T-cells strongly related to lower pathologic grade (Figure 7E). Survival analyses revealed that higher proportions of TILs (resting memory CD4 T-cells and activated NK cells), and lower proportions of eosinophils and activated mast cells, significantly correlated with better 3- or 5-year survival rates (Figure 7F). A TIICs-based regulatory schematic was used to illustrate potential interactions between prognostic TIICs, IRG panel, and stromal cell content-related DEG panel. Most poor prognosis-associated genes (12/18) correlated negatively with favorable prognostic TILs (resting memory CD4 T-cells and activated NK cells) (Figure 7G). We speculate these prognostic genes remodel TIME, leading to differential immune cell infiltration, thereby influencing HCC clinical outcomes.

## DISCUSSION

Multiple studies have subtyped HCC using genomics, transcriptomics, and metabolomics [24–26]. However, few have examined HCC classification by immune signatures. Here, we identified immune-related HCC subtypes using immunogenomic profiles, stromal cell features, and immune cell composition. Our data show that intratumoral genetic and immune microenvironment heterogeneities are essential features of HCC. Understanding individualized immune signature as a potential avenue is expected to improve immunotherapeutic responsiveness.

Tumor gene expression profiling has identified gene expression signatures with prognostic value that can inform patient stratification for targeted therapies. Recent studies have evaluated the expression pattern of IRGs in solid tumors patients receiving immune-based therapies. For instance, in samples from melanoma patients receiving recombinant IL2 treatment, a signature that could predict the clinical response was identified [27]. Elsewhere, an IFN-inflammatory immune gene expression-based signature, which correlated with progression-free survival and enhanced overall response rates, was established in melanoma patients receiving pembrolizumab. The signature is also being investigated in other malignancies [28]. T-effector/IFNγ signature, a 8-gene signature reflecting preexisting immunity, is in phase II trial on previously treated non-small cell lung carcinoma (NSCLC) [29]. Here, we classified HCC into 2 stable subtypes (Immunity High and Immunity Low) based on ssGSEA score. From 729 genes exhibiting differential expression between subtypes, a panel of 6 IRGs was found to significantly correlate with survival outcome, with high prognostic value. Moreover, expression of individual genes in this panel, including IL18RAP, CXCL11, FCER1G, CSF3R and IL2RG were strongly linked to distant metastasis, lymph node metastasis, tumor stage, clinical stage or pathologic grade. In Immunity_H subtype, HLA genes, PD-L1 protein, chemotactic factors related to immunologic activity, MHC-class II protein complex, natural killer cell chemotaxis, antigen processing and presentation were upregulated. This could lead to favorable clinical outcomes. The positive correlation between the prognostic IRG panel-based risk score and TMB, MSI or MMR genes expression also strongly suggested that patients in the high risk group might benefit more from immunotherapy. Thus, identification of intratumoral heterogeneity with regards to prognostic IRG panel, immune-related cellular component, biological process and pathways are used to build prognostic models of pathologic grade, TNM stages, and survival. It may also guide the selection of potential immunotherapeutic targets and individualize treatments.

Tumors possess a stromal compartment comprising cellular and non-cellular components, which also influence cancer development, progression, and metastasis [30, 31]. Although studies have shown that anti-cancer therapies target cancer cells, their effect on the tumor stroma has not been well defined. As the 2 main types of non-tumor components in tumor immune microenvironment, stromal cells and immune cells are considered necessary in cancer diagnostic and prognostic applications [32]. In bulk urothelial cancer transcriptomes, Li Wang et al. recently reported that the primary source of EMT-related gene expression is the non-hematopoietic stromal cells. In a study sample comprising patients with metastatic urothelial cancer under nivolumab treatment, higher EMT/stroma-associated genes expression were related to lower response rate, shorter progression-free survival and poorer overall survival in patient tumors with T-cell infiltration [33]. Here, we grouped HCC into high stromal cell content group (Stroma cell_H), low stromal cell content group (Stroma cell_L), high immune cell content group (Immunecell_H) and low immune cell content group (Immune cell_L) based on stromal score or immune score and evaluated stromal component-related pathogenesis. Our data show that Stroma cell_H correlates with improved 3-year survival significantly. A panel of 17 stromal cell content related DEGs between Stroma cell_H and Stroma cell_L was found to correlate with survival rate significantly. Moreover, the expression level of individual genes in this panel, including GBP5, IL7R, PNMA2, ITGA3, COL16A1, FCGR1A, MEP1A, CTSV, FAM183A and PLAC8, were strongly associated with distant metastasis, lymph node metastasis, tumor stage, clinical stage, or pathologic grade. A positive correlation between risk score based on stromal cell content-related prognostic genes and MSI or MMR genes expression also strongly showed that the patients in the high risk group are more likely to benefit from immunotherapy. These data reflect high heterogeneity of tumor stromal cells in HCC, suggesting that anti-cancer therapies should not only target cancer cells but also the stromal compartment for effective outcomes.

The terms ‘cold’ and ‘hot’ refer to non-inflamed tumors, inflamed but non-infiltrated, and T cell-infiltrated, reflecting lower and higher Immunoscore categories [34]. Hot tumors are characterized by the presence of TILs, expression of anti-PD-L1 on tumor-associated immune cells, potential genomic instabilities and existence of a previous anti-tumor immune response [35–37]. These tumors had a higher response to immune-based therapy. Here, we show that multiple immune cell types were present at significantly higher abundance in Immunity_H, especially the TILs, including memory B-cells, naive B-cells, activated CD4 memory T-cells, CD8 T-cells, follicular helper T cells, and gamma delta T-cells. Higher proportions of TILs significantly correlated with improved 3-, 5- or 10-year survival rates. Moreover, Immunity_H displayed significantly elevated PD-L1 level relative to Immunity_L. Given that TILs are relatively rare in HCC [38], our study highlights immune cell heterogeneity in HCC, suggesting a novel way of stratifying patients for immunotherapy. Our TIICs-based regulatory schematic also highlights avenues for converting immune cold tumors into hot tumors.

Immunophenotypes of solid tumors fall into 3 subtypes: inflamed, immune excluded, and immune desert [32]. 3 distinct immune-cell infiltration subtypes have recently been identified [39]. Relative to previously reported immune subtypes, our clustering based on ssGSEA analysis identified only 2 subtypes. This may arise from the fact that our study focus on the prognostic value. The two subtypes, Immunity_H group and Immunity_L group, exhibited distinct survival rates in both the TCGA and GSE14520 datasets (Figure 1H). However, the prognostic value of Immunity_M group was not consistent across datasets when samples were grouped into 3 groups (data not shown), indicating that 2 subtypes are more effective and that Immunity_H group predicts better survival. In some studies, classification was mainly based on immune-cell infiltration. Here, we took both immune-cell infiltration and immune-related pathways into consideration. This is because high immune-cell infiltration does not necessarily mean high immune response because cells may not be activated. Thus, our strategy may more accurately reflect the immune microenvironment landscape.

Given the significance of HCC immune landscape, our study improves our understanding of intratumoral genetic and immune microenvironment heterogeneity from tumor and non-tumor components perspectives. Here, we reveal potential molecular mechanisms and approaches to manipulate the immune status to improve individualized immunotherapy.

## MATERIALS AND METHODS

### Data acquisition and clinical samples

FPKM-normalized RNA-sequencing data from 369 primary HCC cases and corresponding prognostic data were retrieved from TCGA (https://https://portal.gdc.cancer.gov).

### Clustering

Enrichment level and activity of several immune cells, pathways or functions in HCC were analyzed using single sample gene-set enrichment analysis (ssGSEA) score based on 29 immune-associated gene sets [12]. Hierarchical clustering of HCC was done based on the ssGSEA scores of the 29 immune signatures using the “sparcl” package on R.

### Estimation of immune score, stromal score, and tumor purity

The normalized expression matrix was analyzed by ESTIMATE and the immune score (the infiltration level of immune cells), stromal score (the level of stromal cells present) and tumor purity for each HCC sample determined [40].

### Identification of differentially expressed genes

Differentially expressed genes were identified using “limma”, “ggpubr” and “pheatmap” packages, with FDR =<0.05 and a log2 |fold change| >1 as cutoffs.

### Gene-set enrichment analysis

Functional enrichment analyses via GO term and KEGG pathway analyses, were performed using GSEA (R implementation) [41–43].

### Identification of immune-related gene

IRGs were identified using ImmPort (immunology database and analysis portal) database [44]. These genes are known to be involved in immune activity. Differentially expressed IRGs were extracted from all differentially expressed genes.

### Identification of survival-associated genes and survival analyses

Survival-associated genes were selected using univariate Cox’s proportional hazards regression analysis. Kaplan-Meier analysis was used to compare survival differences. Log-rank test was used to calculate statistical significance with *p =* <0.05 as the threshold.

### Identification of gene-based prognostic index (GPI)

Survival-associated genes were subjected to multivariate Cox’s regression analyses, with integrated genes panels as independent prognostic indicators for GPI development. Immune-related gene-based prognostic index (IRGPI) was based on expression data multiplied by the Cox regression coefficient. Patients were divided into high- and low-risk groups based on the median risk score.

### Construction of transcription factors (TFs) regulatory network

A list of 318 TFs was obtained from the Cistrome cancer database which integrates cancer genomics data from TCGA with over 23000 ChIP-seq chromatin profiling data from Cistrome, illuminating regulatory links between TFs and the transcriptome [45].

### Evaluation of the tumor-infiltrating immune cells (TIICs) proportion

Normalized gene expression data were used to estimate the fraction of 22 infiltrating immune cell types using CIBERSORT as previously described [22]. 1000 CIBERSORT permutations and cases were set with *p*= <0.05 as a cutoff value. Mann-Whitney U test was used to compare the proportion of immune cell subsets between HCC subtypes.

### TMB analysis

Somatic mutation data on 357 hepatocellular carcinoma patients were obtained and processed using VarScan software from the “Masked Somatic Mutation” category on TCGA. Next, TMB was defined and calculated using the formula: (total count of variants)/(the whole length of exons). Detected variants included base substitutions, deletions, or insertions.

### MSI analysis

MSI status for 367 HCC cases consecutively sequenced with Memorial Sloan Kettering-Integrated Mutation Profiling of Actionable Cancer Targets clinical NGS assay was determined using MSI sensor algorithm, a program that reports the percentage of unstable microsatellites [46].

### Statistical analysis

To examine performance of the prognostic index, time-dependent receiver operating characteristic (ROC) curve was calculated using “survivalROC” package [47]. Multivariate Cox regression analyses were performed to verify if the risk score was an independent prognostic index. All analyses were performed on R and the regulatory network visualized using Cytoscape software version 3.7.1 (https://cytoscape.org/). Statistical tests were two-sided. *p* = <0.05 indicated statistical significance.

## Supplementary Material

Supplementary Figure 1
