# Supplementary material for: Identification of immune landscape signatures associated with clinical and prognostic features of hepatocellular carcinoma
Source: Aging (Albany NY). 2020 Oct 13;12(19):19641–59. doi: 10.18632/aging.103977 (PMC7732284; doi:10.18632/aging.103977)
Supplement: Supplementary Figure 1 [file aging-12-103977-s001.pdf]

SUPPLEMENTARY FIGURE

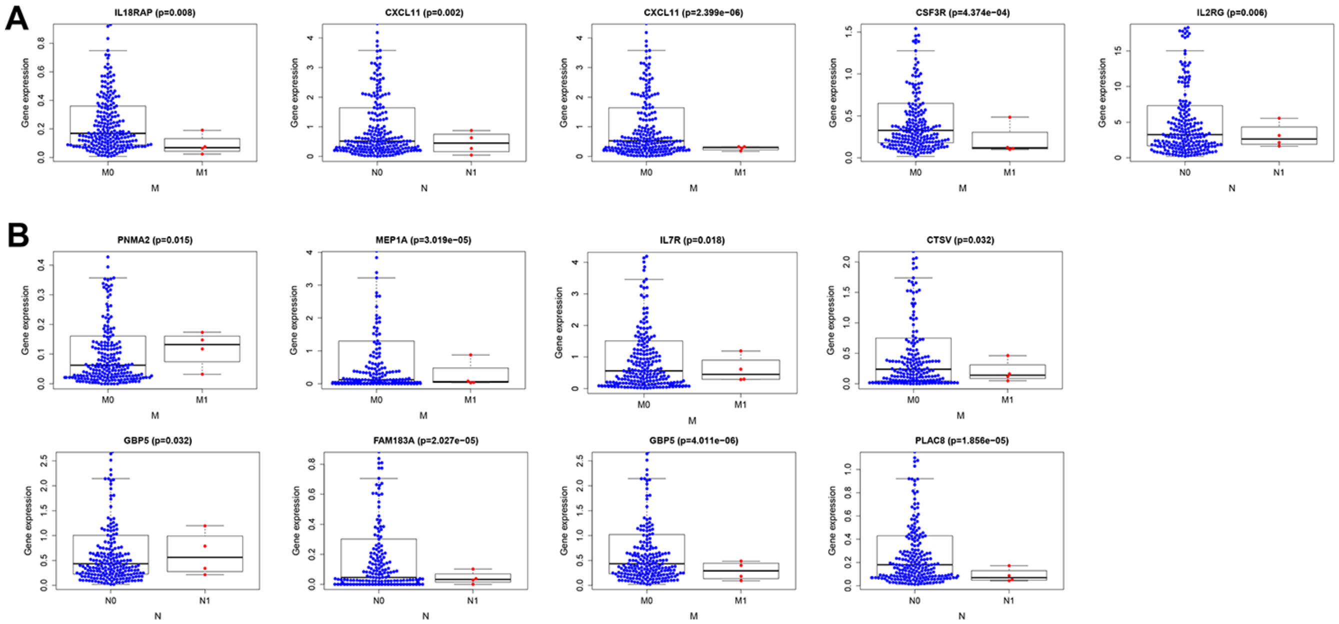

Supplementary Figure 1. Clinical utility of the prognostic IRG panel and the stromal cell content-related prognostic DEG panel. (**A**, **B**) Relationships between genes in prognostic IRG panel (**A**) or stromal cell content-related prognostic DEG panel (**B**) and HCC clinicopathological features (lymph node metastasis status and distant metastasis status).
